# Adult granulosa cell tumor of the ovary incidentally discovered and ruptured during cesarean section: A case report

**DOI:** 10.1097/MD.0000000000045416

**Published:** 2025-10-24

**Authors:** Yayuan Zhou, Linling Zhu, Wenhui Wang, Yujie Hu, Haiyan Wen

**Affiliations:** a Department of Obstetrics, Hangzhou Women’s Hospital (Hangzhou Maternity and Child Health Care Hospital), Hangzhou, Zhejiang, China.

**Keywords:** cesarean section, cyst rupture, granulosa cell tumor of the ovary, pathological diagnosis, pregnancy

## Abstract

**Rationale:**

Ovarian granulosa cell tumor is a rare sex cord-stromal malignancy (2%–5% of ovarian carcinomas). Its diagnosis and management become particularly challenging when this tumor is associated with pregnancy and complicated by intraoperative rupture.

**Patient concerns:**

A 40-year-old multiparous woman underwent emergency cesarean section for fetal distress at 39 weeks. A previously unidentified 2 cm left ovarian cyst was discovered and incidentally ruptured during cystectomy.

**Diagnoses:**

Histopathology analysis revealed the diffuse nests of monomorphic cells exhibiting nuclear grooves and Call-Exner bodies. Immunohistochemistry analysis was positive for FOXL2, inhibin, and vimentin, confirming adult granulosa cell tumor classified as International Federation of Gynecology and Obstetrics IC1.

**Interventions:**

Initial cystectomy was performed. The patient subsequently underwent a postpartum fertility-sparing, including laparoscopic staging procedure, which included left salpingo-oophorectomy and omentectomy; no residual disease was identified.

**Outcomes:**

No evidence of recurrence was detected during follow-up. The patient retained fertility potential and declined adjuvant therapy.

**Lessons:**

Systematic adnexal evaluation during cesarean sections is essential for detecting occult ovarian neoplasms, emphasizing the importance of multidisciplinary collaboration and accessible intraoperative frozen section analysis, particularly in resource-limited settings.

## 1. Introduction

Granulosa cell tumor (GCT) of the ovary is a rare subtype of ovarian cancer, originating from the ovarian mesenchyme and sex cords. It accounts approximately 2% to 5% of all malignant ovarian cancers.^[1]^ The estimated incidence of GCT is between from 0.4 to 1.7 per 100,000 women.^[2–4]^ Based on clinical and histological characteristics, GCT is classified into 2 distinct subtypes: adult granulosa cell tumor (AGCT) and juvenile GCT. AGCT is more prevalent than juvenile GCT, accounting for approximately 95% of all GCT cases.^[3]^ The median age at diagnosis for patients with AGCT is between 50 and 55 years, with the majority of women being postmenopausal and multiparous, which makes the co-occurrence of pregnancy and AGCT exceptionally rare.^[5]^ Here we describe a new case of pregnancy-associated AGCT complicated by intraoperative rupture during cesarean delivery, indicating the importance of intraoperative exploration and frozen section analysis for guiding surgical decisions.

## 2. Case report

A 40-year-old multipara (gravida 3, para 1) with 39 weeks of gestation was admitted to our hospital due to hyperlipidemia on January 22, 2025. In the past obstetrical and gynecological history of this patient, multiple uterine fibroids for nearly 10 years. The diameter of the largest fibroid before pregnancy was about 2 cm, and the current fibroid was about 4 cm. There was no abnormal vaginal bleeding, fever or other discomfort during pregnancy. Prenatal ultrasound examination revealed no evidence of adnexal masses. On January 23, 2025, the patient was indicated for the cesarean section due to the fetal intrauterine distress and being an elderly mother. A newborn boy of 3480 g was given birth with Apgar scores of 10/1’ to 10/5’. After fetal delivery, adnexal examination revealed a tumor on the left ovary, measuring 2 cm × 2 cm × 2 cm.The right adnexa were normal. While the peritoneum and the omentum were also normal. Then the ovarian cystectomy was performed, during which the cyst was ruptured. Peritoneal lavage was subsequently performed, and the cyst wall was sent for pathological examination. Intraoperative findings revealed a thin-walled, unilocular cyst containing dark red hemorrhagic fluid and decidua-like material.

**Figure 1.**
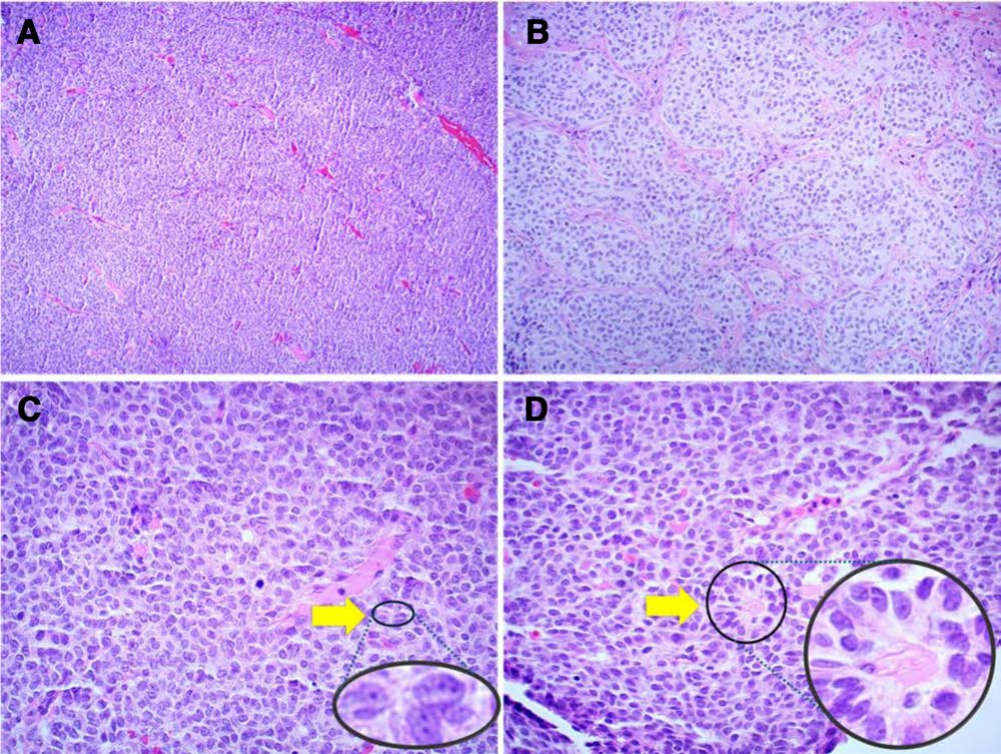
Histological examination of AGCT. (A) Neoplastic cells with diffuse growth, scant cytoplasm, and monomorphic appearance (×40). (B) Tumor cells in nests surrounded by fibrous septa with ill-defined borders (×100). (C) Cells with uniform size, ovoid to polygonal shape, nuclear grooves, and rare mitotic figures (×400). (D) Presence of Call-Exner bodies (×400). AGCT = adult granulosa cell tumor.

**Figure 2.**
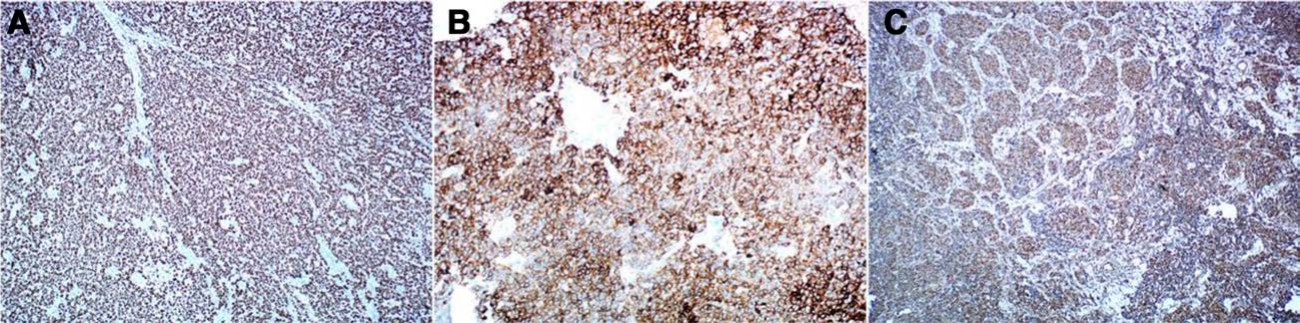
Immunohistochemistry showing positivity for (A) FOXL2 (×40), (B) inhibin, and (C) vimentin.

On microscopic examination, as shown in Figure 1A, the tumor cells are diffusely distributed. The cytoplasm of the tumor cells is relatively scarce and shows a single manifestation. The tumor cells are arranged in nests and clusters, with fibrous septa surrounding them. The boundaries of the tumor cells are indistinct (Fig. 1B). The cytoplasm of tumor cells is scanty, and the sizes of tumor cells are relatively uniform. They are mostly oval or polygonal in shape. Nuclear grooves can be observed, or they may appear as coffee beans. Nuclear division is rare (Fig. 1C). Call-Exner bodies are present (Fig. 1D). Immunohistochemistry was positive for FOXL-2, inhibin and vimentin (Fig. 2A–C), negative for epithelial membrane antigen. The final diagnosis was an AGCT of the ovary. The patient was diagnosed as the International Federation of Gynecology and Obstetrics (FIGO) stage IC1.

Following the return of the pathological findings, a consultation with a tumor specialist was conducted, who recommended comprehensive staging surgery for the patient. However, the patient refused and requested surgery to preserve her fertility. Therefore, 42 days after giving birth (after the postpartum period), a laparoscopic left salpingo-oophorectomy, multiple peritoneal biopsies and partial resection of greater omentum were performed. Postoperative pathology results were all negative. The patient was fully informed of the necessity for follow-up care. After a 3-month follow-up, the patient showed no signs of tumor progression. The study was approved by the Ethical Review Committee and conducted in accordance with the principles of the Declaration of Helsinki. The patient provided written informed consent for publication and the use of her images.

## 3. Discussion

AGCT originates from the proliferation of normal preovulatory granulosa cells and retains some of their characteristics, including the ability to produce and secrete steroid hormones-predominantly estrogen and, to a lesser extent, androgen.^[6]^ The elevated estrogen secretion manifests as the precocious puberty in prepubertal individuals, the menstrual irregularities in reproductive-age women, and the endometrial pathologies in perimenopausal women. And a subset of patients may also present with concurrent endometrial hyperplasia or even carcinoma.^[7]^ The clinical presentation of AGCT is often atypical, with approximately 20% of cases being incidentally detected.^[5]^ Notably, the GCT in this patient exhibited no detectable sonographic abnormalities during preoperative transabdominal ultrasound evaluation. Since the hormonal changes during pregnancy may mask tumor-related symptoms, and the gravid uterus can also obscure the imaging findings, leading to the misdiagnosis or missed diagnoses of ultrasound evaluation. On the other hand, this case highlights the diagnostic limitations of conventional ultrasonography in identifying the ovarian stromal neoplasms with subtle imaging features.^[8]^ This observation aligns with the reported challenges in visualizing AGCTs, which frequently present as isoechoic masses lacking the characteristic calcifications or vascular patterns typical of malignant epithelial ovarian tumors. The absence of discernible tumor boundaries and the minimal posterior acoustic shadowing further contribute to the false-negative imaging interpretation due to their homogeneous cellular architecture and frequent lack of cystic components.^[8–10]^ In previously reported cases, AGCTs always presented as large solid masses, with diameters exceeding 7 cm.^[11–15]^ In this case, the ovarian mass was incidentally discovered during an emergency cesarean section, emphasizing the importance of systematic intraoperative exploration of bilateral adnexa and the pelvic cavity – particularly in patients without comprehensive preoperative imaging evaluation.

To date, there are very few reported cases of AGCT coexisting with pregnancy in the literature, as summarized in Tables 1 and 2. Notably, Kumar et al reported a case of right ovarian AGCT with rupture but not during pregnancy or cystectomy – rather, it occurred in the context of torsion presenting as acute abdomen.^[13]^ The patient, in her late 40s and not pregnant, had an ultrasound revealing a large multiloculated right adnexal mass with ascites, prompting further evaluation with MRI. The MRI showed cystic/necrotic regions measuring approximately 16 cm × 14 cm × 11 cm. Histopathology confirmed a well-differentiated AGCT. Imaging findings suggested an ovarian neoplasm with torsion and hemoperitoneum, leading to emergency laparotomy. According to previous case reports, for relatively large tumors of the AGCT, it can be detected during imaging studies like ultrasound and MRI, revealing tumor characteristics such as solid-cystic masses and heterogeneous echotexture. However, a thorough and methodical intraoperative assessment of both bilateral adnexa and the pelvic cavity is strongly recommended, especially for the early stages of GCT, when the neoplasm is typically small and confined. Furthermore, conservative management is usually prioritized in younger patients to preserve fertility and maintain future reproductive options. More aggressive treatments, such as oophorectomy or chemotherapy, are required in cases exhibiting advanced-stage disease or associated complications. In our case, a small (2 cm) left ovarian cyst was identified and incidentally ruptured during cystectomy. It should be noted that intraoperative rupture increases the risk of iatrogenic tumor dissemination and peritoneal carcinomatosis.^[19]^ AGCT cells implanted in the peritoneum may lead to late recurrences (5–30 years post-surgery), a particular concern given the endocrine activity of the tumor. Other key hazards include potential hemoperitoneum, hemorrhagic shock, and endocrine complications.^[20]^ Rupture automatically upstages the tumor to the FIGO IC1 stage, requiring closer surveillance.^[19]^ In our case, peritoneal lavage was performed to mitigate the risk of tumor cell dissemination and subsequent recurrence following intraoperative rupture.

**Table 1:** Circumstances of discovery.

Author and year	Age of patient	Circumstances of discovery	Pregnancy and fetal outcomes	Birth weight (g)
Guidi et al, 2021^[15]^	41	Recurrent, 29 wk	Pregnancy, live birth, female	2750
Fernandez-Cid et al, 2010^[14]^	35	Tumor detected at routine ultrasound, 15 wk gestation	Pregnancy, live birth, male	Not mentioned
*Aymen et al, 2016^[16]^	30	Incidentally discovered a 40 cm × 30 cm ruptured tumor attached to the right ovary during CS, 32 wk gestation	Pregnancy, live birth	1925
Kaakoua et al, 2015^[11]^	32	Tumor detected during primary infertility evaluation	Nulliparous and nulligravida	Not mentioned
Kumar et al, 2024^[13]^	Late 40s	Tumor detected in Pelvic examination due to sudden right iliac fossa pain, vomiting and 1 febrile episode	Multiparous and nulligravida	Not mentioned
Pham et al, 2023^[12]^	44	Incidentally discovered during CS, 39 wk gestation	Pregnancy, live birth, male 3000	3000
Roy et al, 2015^^[17]^^	23	Incidentally discovered during CS, full-term pregnancy	Pregnancy, live birth, male	2500
*Agarwal et al, 2011^^[18]^^	26	Recurrent, patient with acute abdominal pain and a ruptured 9.7 cm × 7.7 cm ovarian tumor, ascites, peritoneal implants, and pleural effusion, 20 wk gestation	Pregnancy, live birth, male	1200
Current case	40	Incidentally discovered during CS, 39 wk	Pregnancy, live birth, male	3480

The cases involving tumor rupture during pregnancy are marked with asterisk (*).

CS = cesarean section, TP = termination of pregnancy.

**Table 2:** Management and maternal outcomes.

Author and year	Interventions	Outcomes
Guidi et al, 2021^[15]^	Total hysterectomy, left salpingo-oophorectomy, peritoneum biopsy Interventions during CS. + Six cycles with carboplatin after TP	No recurrence after 26 months follow-up
Fernandez-Cid et al, 2010^[14]^	Not mentioned	Symptomatic and without recurrence on ultrasound
*Aymen et al, 2016^^[16]^^	Right adnexectomy and partial omentum section during CS. +Total hysterectomy + left adnexectomy + Appendicectomy + Peritoneum biopsy + 4 cycles of BEP protocol after TP.	No recurrence after 18 months follow-up
Kaakoua et al, 2015^[11]^	Conservative surgery including left adnexectomy and infra-colonic omentectomy with peritoneal biopsies, and peritoneal cytology followed by 3 cycles of adjuvant chemotherapy every 3 wk.	No recurrence after 5 years of follow-up
Kumar et al, 2024^[13]^	The patient was advised to do a postoperative inhibin B and referred to a dedicated oncology center for chemotherapy and follow-up.	Not mentioned
Pham et al, 2023^[12]^	Left oophorectomy during CS. Four-cycle regimen of Carboplatin and Paclitaxel after TP.	No recurrence after 32 months follow-up
Roy et al, 2015^^[17]^^	Right ovariectomy during CS. Chemotherapy was used, but the type of drug used was NS after TP.	Not mentioned
*Agarwal et al, 2011^[18]^	Adriamycin-Vincristine at 21 wk during pregnancy. +Total hysterectomy, +left salpingo-oophorectomy, +six cycles of cisplatin after TP.	No recurrence after 10 months follow-up
Current case	+ovarian cystectomy during CS; +laparoscopic left salpingo-oophorectomy, +multiple peritoneal biopsy, +partial resection of greater omentum After TP.	No recurrence after 3 months follow-up

The cases involving tumor rupture during pregnancy are marked with asterisk (*).

CS = cesarean section, TP = termination of pregnancy.

The diagnosis of AGCT relies on pathological examination which typically reveals characteristic Call-Exner bodies under microscopy (see Fig. 2B) and immunohistochemical detection of the FOXL2 mutation – a hallmark feature of AGCT.^[21]^ Intraoperative frozen section analysis can assist the surgical decision-making; however, in this case, the unavailability of nighttime frozen pathology services at our specialized hospital delayed the clinical decision-making. To optimize patient management, the process improvements such as establishing regional pathology collaboration should be implemented to ensure timely and accurate intraoperative diagnosis.

The evidence-based management of AGCT remains challenging due to the rarity of the condition. Currently, no standardized international therapeutic guidelines exist for AGCT, though the FIGO staging system remains the clinical standard.^[22]^ Surgical intervention remains the cornerstone of treatment for both primary and recurrent AGCT, as no alternative therapies have demonstrated comparable efficacy. Comprehensive staging surgery includes omentectomy, systematic abdominal cavity examination, biopsies of the diaphragmatic peritoneum, paracolic gutters and pelvic peritoneum, plus peritoneal washings.^[23]^ For premenopausal patients, fertility-sparing surgery may be considered. As AGCTs are low-grade malignancies that rarely exhibit lymph node metastasis in early stages,^[24]^ some scholars propose that pelvic/para-aortic lymphadenectomy may minimally impact recurrence rates in early-stage AGCT.^[25]^ Thus, lymph node dissection may be omitted during comprehensive staging surgery. However, no consensus exists on the optimal extent of lymphadenectomy in early-stage AGCT, warranting further research to establish standardized protocols.

As previously noted, reports of ruptured AGCTs during pregnancy are exceptionally rare in the medical literature. Non-pregnancy studies show that the key prognostic factor is the FIGO stage at initial surgery.^[26]^ Patients with GCTs exhibiting high-risk features – including age > 50 years, residual tumors, intraoperative rupture, CA-125 levels (≥35 IU/mL), large tumor size, and diabetes – have a higher recurrence risk and should be evaluated for adjuvant chemotherapy.^[6,25,27]^ The 5-year disease-specific survival rates for AGCTs are 98% (stage I), 84% (stage II), 61% (stage III), and 41% (stage IV).^[28]^ GCTs frequently demonstrate late and multiple recurrences.^[23]^ GCTs recur in 20% of stage I and 43% to 48% of advanced-stage (II–IV) cases. Postoperative relapse typically occurs at a median of 4 to 6 years, with recurrences reported up to 20 years later, indicating the necessity of long-term surveillance. The 3-month follow-up period in our study reflects the initial phase of patient monitoring in this study, for the purpose of assessing the immediate outcomes. We are committed to continued follow-up of the patient as part of future research efforts. Postoperative management requires long-term follow-up, particularly with ovarian preservation, including systematic intraoperative exploration of both bilateral adnexa and the pelvic cavity, along with regular inhibin B monitoring and ultrasound examinations.

The incidental discovery during cesarean section demonstrates both the limitations of prenatal imaging surveillance and the need for refined diagnostic algorithms in high-risk pregnancies. And it also highlights the critical intraoperative management dilemmas, particularly balancing tumor resection against maternal-fetal safety during emergency cesarean delivery. Finally, it emphasizes the necessity of multidisciplinary collaboration (obstetrics, gynecologic oncology, and pathology) to optimize outcomes, especially considering the tumor’s endocrine activity and estrogen-driven complications. In the future, it is crucial to improve the clinical protocols of pregnancy-associated ovarian neoplasms and deepen the understanding of tumor biology in hormonally dynamic environments are essential.

## 4. Conclusion

This case report describes the incidental intraoperative rupture of a small (2 cm) AGCT that was not detectable on preoperative transabdominal ultrasound, highlighting the diagnostic limitations of conventional imaging for ovarian stromal tumors. Intraoperative findings emphasized the importance of systematic exploration of the bilateral adnexa and pelvic cavity. Frozen section analysis played a key role in guiding immediate surgical decisions. The case also reflects the practical challenges of managing ruptured ovarian tumors in the context of advanced maternal age and limited resources in a primary care hospital setting. The 3-month follow-up in the study assessed early outcomes, but AGCTs carry a risk of late recurrence over 5 to 30 years, indicating the need for long-term surveillance, which we will aim to continue in our future research.

## Author contributions

**Conceptualization:** Yayuan Zhou.

**Data curation:** Linling Zhu, Wenhui Wang, Yujie Hu.

**Supervision:** Haiyan Wen.

**Writing – original draft:** Yayuan Zhou.
